# Concurrent coronary artery disease and immune thrombocytopenia: a systematic review

**DOI:** 10.3389/fmed.2023.1213275

**Published:** 2023-10-10

**Authors:** Alaa Rahhal, Drew Provan, Khaled Shunnar, Mostafa Najim, Ashraf Omer Ahmed, Waail Rozi, Murtadha Al-Khabori, Mahmoud Marashi, Mona AlRasheed, Hani Osman, Mohamed Yassin

**Affiliations:** ^1^Pharmacy Department, Hamad Medical Corporation, Doha, Qatar; ^2^Barts and The London School of Medicine, Queen Mary University of London, London, United Kingdom; ^3^Cardiology Department, Hamad Medical Corporation, Doha, Qatar; ^4^Internal Medicine Department, Rochester Regional Health—Unity Hospital, New York, NY, United States; ^5^Internal Medicine Department, Hamad Medical Corporation, Doha, Qatar; ^6^Hematology Department, Sultan Qaboos University, Muscat, Oman; ^7^Dubai Academic Health Corporation and Mediclinic Hospital, Dubai, United Arab Emirates; ^8^Hematology Department, AlAdan Hospital, Hadiya, Kuwait; ^9^Hematology and Oncology Department, Tawam Hospital, Abu-Dhabi, United Arab Emirates; ^10^Hematology Department, National Centre for Cancer Care and Research, Hamad Medical Corporation, Doha, Qatar

**Keywords:** coronary artery disease, acute coronary syndrome, immune thrombocytopenia, intravenous immunoglobulins, percutaneous coronary intervention, coronary artery bypass graft surgery

## Abstract

**Introduction:**

Coronary artery disease (CAD) management in the setting of immune thrombocytopenia (ITP) remains very challenging to clinicians as a reasonable balance between bleeding and thrombosis risks needs to be achieved, and the evidence guiding such management is scarce.

**Methods:**

We conducted a systematic review following the PRISMA guidelines to summarize the available literature on the management and outcomes of CAD coexisting with ITP. We searched PubMed and Embase for studies published in English exploring CAD and ITP management until 05 October 2022. Two independent reviewers screened and assessed the articles for inclusion. Patients' characteristics, CAD treatment modalities, ITP treatment, and complications were reported.

**Results:**

We identified 32 CAD cases, among which 18 cases were revascularized with percutaneous coronary intervention (PCI), 12 cases underwent coronary artery bypass graft surgery (CABG), and two cases were managed conservatively. More than 50% were men, with a mean age of 61 ± 13 years and a mean baseline platelet count of 52 ± 59 × 10^9^/L. Irrespective of the revascularization modality, most patients were treated with either corticosteroids alone, intravenous immunoglobulins (IVIG) alone, or in combination. Among those who underwent PCI, two patients had bleeding events, and one patient died. Similarly, among those with CABG, one patient developed bleeding, and one patient died.

**Conclusion:**

We found that revascularization with either PCI or CABG with the concurrent use of corticosteroids and/or IVIG for ITP was feasible, with an existing non-negligible risk of bleeding and mortality.

## Introduction

Immune thrombocytopenia (ITP) is an acquired autoimmune disorder characterized by a low platelet count due to platelet destruction and impaired platelet synthesis. The incidence of ITP is estimated to be 2–5 per 100,000 persons in the general population and can present as an isolated primary condition or secondary to other conditions (1).

Unstable angina (UA), acute non-ST-elevation myocardial infarction (NSTEMI), and acute ST-elevation myocardial infarction (STEMI) are the three presentations of acute coronary syndromes (ACS). They are considered cardiac emergencies, requiring prompt interventions, including revascularization with percutaneous coronary intervention (PCI), thrombolytic therapy, or coronary artery bypass graft surgery (CABG). Dual antiplatelet therapy (DAPT) consisting of aspirin and a P2Y12 receptor antagonist is the cornerstone of ACS management, with longstanding endorsements by international guidelines, including the American College of Cardiology Foundation (ACCF), the American Heart Association (AHA), and the European Society of Cardiology (ESC) (2, 3). While DAPT effectively reduces the risks of both stent thrombosis and spontaneous ischemic events, it does so at the cost of an increased bleeding risk (4, 5). The management of antiplatelet therapy in ACS patients with thrombocytopenia, particularly ITP, poses a particular challenge for clinicians, as this population is at a higher risk of both bleeding and, paradoxically, thrombotic events (6). Therefore, long-term clinical outcomes, including bleeding and recurrent ACS, might be difficult to attain in this vulnerable population. Similarly, ITP management with ACS remains difficult as corticosteroids, which are first-line therapy for ITP, are associated with increased bleeding risk when used concurrently with DAPT and pose a concern for myocardial healing when used in the setting of ACS. Moreover, some second-line ITP therapies are associated with an increased risk of thrombosis (1, 7).

To date, the literature guiding clinicians on the management of coronary artery disease (CAD) complicated by ITP remains scarce. Therefore, we conducted a systematic review to summarize the available evidence of ACS management in the setting of ITP to help provide future directions and management strategies for this population cohort.

## Methods

### Eligibility criteria

All experimental studies, observational studies, case series, and case reports published in English exploring the management of CAD complicated by ITP until 05th October 2022 were included. Articles were excluded in the following cases: (1) ACS management reported not in the setting of ITP and vice versa; (2) thrombocytopenia other than ITP; (3) non-clinical outcomes were solely reported; or (4) review articles.

### Search strategy

We performed a systematic review following the Preferred Reporting Items for Systematic Reviews and Meta-Analyses (PRISMA) guidelines. We searched PubMed and Embase databases for studies published in English that explored the management of CAD complicated by ITP. We combined the following search terms: “Immune Thrombocytopenia Purpura” AND “Acute Coronary Syndrome,” OR “Percutaneous Coronary Intervention” OR “Coronary Artery Bypass Graft.” The search included all articles published up to 5 October 2022. The reference lists of the retrieved articles were manually screened.

### Study selection and data extraction

The titles and abstracts of the records identified were screened by two independent reviewers (AR/AA). We excluded records that did not meet our eligibility criteria. All relevant abstracts were retrieved in full text and assessed for inclusion in the final report by the same reviewers. Disagreements were resolved through discussion to reach a consensus among the reviewers. The included articles were tabulated, and a pre-made Excel sheet/spreadsheet was used to extract the following parameters: article's last author, year of publication, ACS type, baseline platelet count, coronary intervention done, type of stent implanted, coronary intervention access, ITP treatment used, antithrombotic therapy used, and complications, including mortality, bleeding, and ITP treatment-induced ACS.

### Objectives and outcomes

The objectives of this systematic review were to characterize the coronary interventions used for revascularization in the setting of ITP, to determine the approaches to antithrombotic therapy and ITP treatment used in the setting of ACS complicated by ITP, and to determine the clinical outcomes of ACS complicated by ITP. The outcomes evaluated were as follows: (1) the frequency of coronary interventions, including PCI, CABG, and conservative management; (2) the frequency of using DAPT; (3) the frequency of using different ITP treatment options, including corticosteroids, intravenous immunoglobulins (IVIG), rituximab, and thrombopoietin receptor agonists (TPO-RAs); (4) complications, including mortality, bleeding, and ITP treatment-induced ACS.

## Results

### Included studies

**Figure 1:**
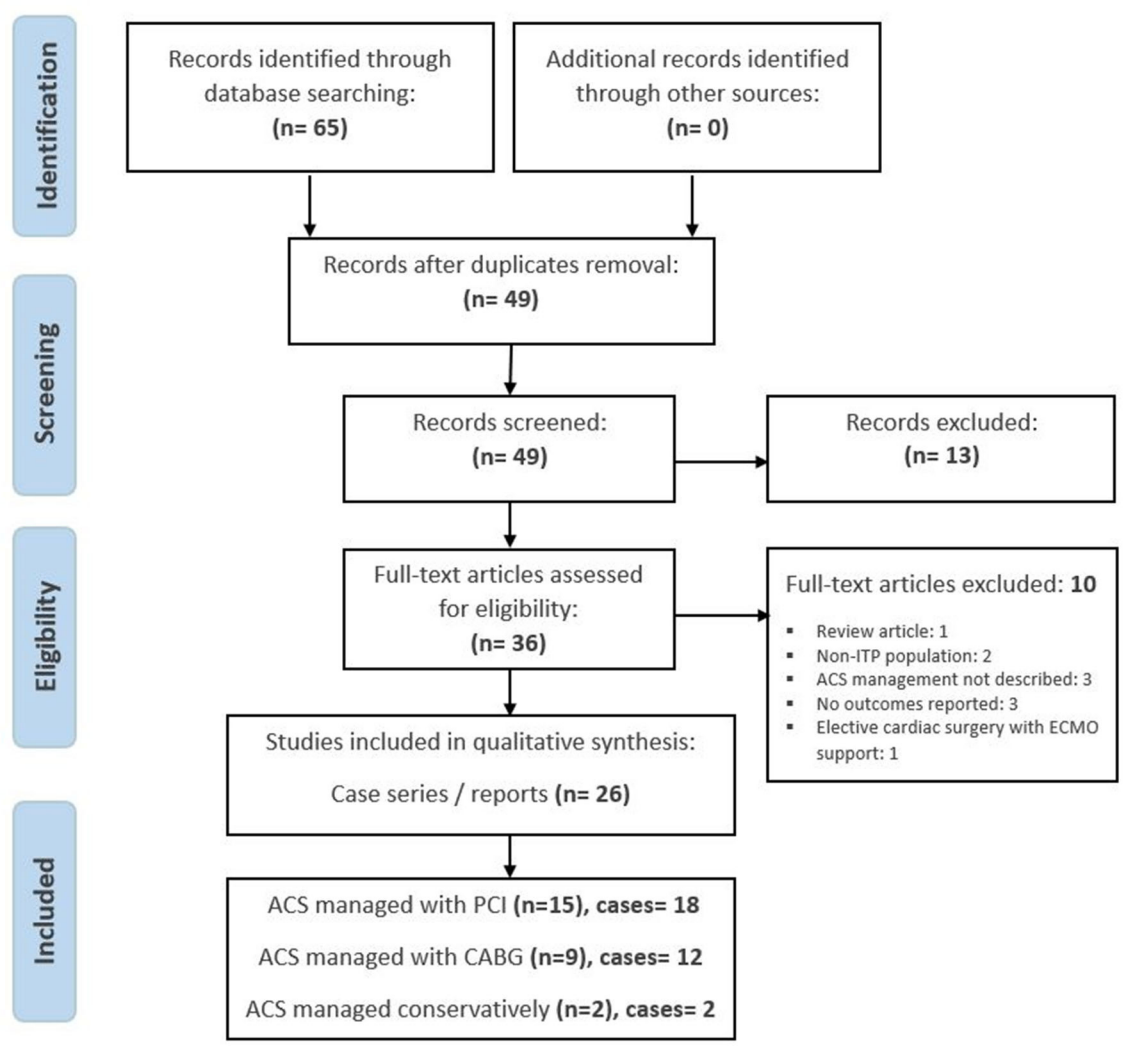
PRISMA flow diagram.

The electronic search identified 65 articles. Of these, 36 were evaluated in full text for eligibility after removing duplicates and non-relevant articles. A total of 10 articles were excluded due to different reasons: ACS management was not described (3), no clinical outcomes were reported (3), non-ITP population (2), review article (1), and intervention used was cardiac surgery-assisted extracorporeal membrane oxygenation (ECMO) support (1). There were 26 case series/reports for a total of 32 cases eligible for inclusion in quantitative analysis, as demonstrated in Figure 1 (8–33). Our net search resulted in 18 cases of CAD complicated by ITP, which were revascularized with PCI (Table 1), 12 cases of CAD that underwent CABG in the setting of ITP (Table 2), and 2 cases treated conservatively without invasive interventions (Table 3).

**Table 1:** Summary of ITP cases presented with CAD and managed with PCI.

**References**	**Age (years)**	**Gender**	**ACS**	**Baseline PLT (× 10^9^/L)**	**Intervention**	**Access (radial, femoral)**	**Stent type**	**Coronary**	**Anticoagulation during PCI**	**ITP treatment**	**Antiplatelet regimen**	**Bleeding**	**Death**	**MI due to ITPtreatment**
Al-Lawati et al. (8)	50	M	STEMI	2 (treated for ITP and readmitted with STEMI with PLT 658)	POBA	Radial	None	RCA	Heparin	• Eltrombobag • IVIG	Aspirin and clopidogrel for 10 weeks then Aspirin for 1 year	No	No	Yes, current STEMI was 1 week post IVIG
Li-Sha et al. (9)	75	M	NSTEMI	16	POBA	Radial	None	LAD	Heparin	• PLT transfusion • Methylprednisolone 1 mg/Kg	Aspirin and clopidogrel	No	No	No
			UA after 4 months	124	PCI	NI	DES	LAD	Heparin	• Methylprednisolone	Aspirin and clopidogrel	No	No	No
Fong et al. (10)	71	F	NSTEMI	16	PCI	Radial	DES	LAD	None	• Dexamethasone 10 mg • IVIG for 5 days	Aspirin and clopidogrel	No	No	No
Fuchi et al. (11)	72	F	NSTEMI	59	POBA	Femoral	None	LAD	Heparin	• PLT transfusion • Methylprednisolone 1,000 mg • IVIG for 5 days	None, except for ethyl icosapentate as antiplatelet	No	No	Yes, re-infarction requiring POBA within a few hours of steroids
Gracia et al. (12)	37	M	STEMI	39	PCI	Femoral	DES	LAD	Heparin	• None	Aspirin and Clopidogrel	No	No	No
Hak et al. (13)	55	M	UA	33	PCI	Radial	DES	LAD	Heparin	• PLT transfusion • Prednisolone 30 mg	Aspirin and clopidogrel for 1 month	Ecchymosis requiring DAPT interruption for a week	No	No
Ikovis et al. (14)	53	M	STEMI	55	PCI	Femoral	DES	LAD	Bivalirudin	• IVIG for 3 days • Methylprednisolone 60 mg followed by 40mg • Romip lostim	Aspirin and Clopidogrel for 4 days, then continued on clopidogrel only	No	Yes, due to pneumonia	No
Kim et al. (15)	47	F	STEMI	21	PCI	Femoral	NI	RCA	Heparin	• IVIG	Aspirin and clopidogrel	No	No	No
Marques et al. (16)	54	M	UA	15	PCI	Brachial	BMS	LCx	Heparin	• IVIG • Methylprednisolone	None	No	No	No
Moretti et al. (17)	66	M	UA	110	PCI	Femoral	BMS and DES	LCx, RCA, LM, and LAD	NI	• Prednisolone	Aspirin and clopidogrel for 6 months then Aspirin alone	No	No	No
Neskovic et al. (18)	80	M	STEMI	5	PCI	Femoral	BMS	LAD	Heparin (received fondaparinux periprocedural; 1 day before and 2 days after PCI)	• Danazol • Prednisolone 60 mg	Aspirin and clopidogrel	No	No	No
Park et al. (19)	61	F	UA	4	PCI	Femoral	BMS	LAD and LCx	NI	IVIG for 3 days	None	No	No	No
Stouffer et al. (20)	77	M	UA	70	POBA	NI	None	LCx	Heparin	None	Aspirin	No	No	No
			NSTEMI after 5 weeks	78	PCI	NI	BMS	LCx	None, but was given eptifibatide	Prednisolone 1 mg/Kg	Aspirin and clopidogrel for 3 weeks then clopidogrel stopped due to bleeding	Diffuse petechiae and a spontaneous nose bleed	No	No
Torbey et al. (21)	61	F	STEMI	322	PCI	Femoral	DES	LAD	Heparin	• None	Aspirin and clopidogrel	No	No	No
	55	M	NSTEMI	42	PCI	Femoral	DES	OM	NI	• IVIG • Prednisolone	Aspirin and clopidogrel	No	No	No
Yildiz et al. (22)	23	F	STEMI	35	PCI	Femoral	BMS	LAD	Heparin	• Chronic steroids	Aspirin and clopidogrel	No	No	No

PCI, percutaneous coronary intervention; CABG, coronary artery bypass graft; CAD, coronary artery disease; STEMI, ST-elevation myocardial infarction; NSTEMI, Non ST-elevation myocardial infarction; UA, unstable angina; PLT, platelet; IVIG, intravenous immunoglobulins; MI, myocardial infarction; ITP, immune thrombocytopenia purpura; LM, left main; LAD, left anterior descending; RCA, right coronary artery; LCx, left circumflex; OM, obtuse marginal; POBA, percutaneous old balloon angioplasty; DES, drug eluting stent; BMS, bare metal stent; NI, no information.

**Table 2:** Summary of ITP cases presented with CAD and managed with CABG.

**References**	**Age (year)**	**Gender**	**CAD**	**Baseline PLT (× 10^9^/L)**	**CABG indication**	**Pre-OP PLT (× 10^9^/L)**	**ITP treatment**	**Procedure (on-pump, off-pump)**	**Antiplatelet regimen (med and duration)**	**Bleeding**	**Death**	**MI due to ITP treatment**
Thompson et al. (23)	61	M	Stable CAD	68	Failed PCI	84	PLT transfusion	On-pump	NI	Yes, surgical site requiring re-exploration	No	No
Koike et al. (24)	37	M	UA	8	MVD	NI	PLT transfusion	On-pump	NI	No	No	No
Briffa et al. (25)	69	M	UA	63	Restenosis after PCI	64	Prednisolone, IVIG × 5 days	On-pump	NI	No	No	No
Mathew et al. (26)	72	M	STEMI	40	MVD	57	IVIG × 2 days, PLT transfusion 7 units	On-pump	NI	No	No	No
	72	F	UA	49	MVD	168	IVIG × 2 days	On-pump	NI	No	No	No
	69	M	STEMI	65	MVD	87	IVIG × 2 days, PLT transfusion	On-pump	NI	No	No	No
Köner et al. (27)	59	M	UA	88	MVD	138	Prednisolone, IVIG × 5 days	On-pump	NI	No	No	No
Inoue et al. (28)	60	F	UA	42	MVD	187	IVIG × 4 days	Off-pump	NI	No	No	No
Fatimi et al. (29)	54	F	Stable CAD	100	MVD	135	Prednisolone	On-pump	NI	No	No	No
Rossi et al. (30)	47	M	NSTEMI	55	MVD	NI	None	On-pump	NI	No	No	No
Chowdhry et al. (31)	55	M	Stable CAD	15	MVD	64	Prednisolone, IVIG × 5 days	On-pump	NI	No	Yes, day 29 post-operation due to bloody cardiac tamponade	No
Torbey et al. (21)	61	M	NSTEMI	23	MVD	180	Dexamethasone and rituximab for 1 then changed to IVIG and dexamethasone	Off-pump	Aspirin 325 mg	No	No	Yes, current NSTEMI was after 1 day of Dexamethasone and rituximab

CABG, coronary artery bypass graft; CAD, coronary artery disease; STEMI, ST-elevation myocardial infarction; NSTEMI, non ST-elevation myocardial infarction; UA, unstable angina; PLT, platelet; IVIG, intravenous immunoglobulins; MI, myocardial infarction; ITP, immune thrombocytopenia purpura; PCI, percutaneous coronary intervention; MVD, multivessel coronary artery disease.

**Table 3:** Summary of ITP case(s) presented with CAD and managed conservatively.

**References**	**Age (year)**	**Gender**	**ACS**	**Baseline PLT (× 10^9^/L)**	**Intervention**	**Anticoagulation**	**ITP treatment**	**Antiplatelet regimen**	**Bleeding**	**Death**	**MI due to ITP meds**
Argawal et al. (32)	67	M	NSTEMI	< 10	Conservative due to low PLT	None	Steroids, IVIG	None	No	No	Yes, developed the current NSTEMI after 3rd dose IVIG
Tabata et al. (33)	75	F	NSTEMI (vasospasm)	5	Conservative as it was vasospasm	None	None	None	No	No	NA

**NSTEMI:** Non ST-Elevation Myocardial Infarction; **PLT:** Platelet; **IVIG:** Intravenous Immunoglobulins; **ITP:** Immune Thrombocytopenia Purpura.

### Characteristics of CAD and ITP cases managed with PCI

The mean age of those with PCI was 61 ± 15 years, and around 70% were men. Seven of them presented with STEMI, five presented with NSTEMI, and the remaining patients had UA. The ACS presentation of the 18 cases required revascularization; 14 had stent implantation, while the remaining patients underwent percutaneous old balloon angioplasty (POBA). Interestingly, more than 50% of patients had their PCI done through the femoral artery. At the time of presentation with ACS, the mean baseline platelet count was 58 ± 75 × 10^9^/L. Only 17% did not receive treatment for ITP, while the remaining 83% received ITP treatment as follows: 39% corticosteroids alone, 28% corticosteroids and IVIG, and 17% IVIG alone. Post-PCI, 78% of patients received DAPT with aspirin and clopidogrel, of whom 28% had to receive DAPT for < 12 months due to thrombocytopenia. Approximately 17% did not receive any antithrombotic therapy post-PCI, and only one patient was treated with aspirin alone.

### Characteristics of CAD and ITP cases managed with CABG

Twelve patients underwent CABG with a mean age of 60 ± 10 years, and three-quarters of them were men. Half of them presented with UA, while the remaining 50% had STEMI, NSTEMI, and stable angina with positive stress tests that were equally distributed, as shown in Table 4. Upon presentation, the mean baseline platelet count was 51 ± 28 × 10^9^/L and 116 ± 51 × 10^9^/L pre-operatively, respectively. One patient did not receive treatment for ITP, two patients received platelet transfusion only, one patient received corticosteroids alone, four patients received corticosteroids and IVIG, and four patients were treated with IVIG alone. Antithrombotic therapy details post-CABG were not reported in 92% of the cases.

**Table 4:** Combined characteristics of ITP cases vascularized with different modalities.

**Characteristics**	**PCI, *n* (%) (*n* = 18)**	**CABG, *n* (%) (*n* = 12)**	**Conservative, *n* (%) (*n* = 2)**
Age (years)	61 ± 15	60 ± 10	71 ± 6
Male gender	12 (67)	9 (75)	1 (50)
**CAD**			
STEMI	7 (39)	2 (17)	0
NSTEMI	5 (28)	2 (17)	2 (100)
UA	6 (33)	6 (50)	0
Stable CAD	0	2 (17)	0
**Baseline PLT (**×**10**^9^**/L)**			
Mean ± SD	58 ± 75	51 ± 28	7.5 ± 3.5
Minimum	2	8	5
Maximum	322	100	10
**ITP Treatment** ^*^			
Steroids	7 (39)	1 (8)	0
IVIG	3 (17)	4 (33)	0
Steroids and IVIG	5 (28)	4 (33)	1 (50)
None	3 (17)	1 (8)	1 (50)
**Antithrombotic therapy**			
DAPT < 12 months	5 (28)	0	0
DAPT with unspecified duration	9 (50)	0	0
Aspirin alone	1 (6)	1 (8)	0
None	3 (17)	0	2 (100)
No information	0	11 (92)	0
**Complications**			
Bleeding events	2 (11)	1 (8)	0
Death	1	1 (8)	0
MI due to ITP treatment	2 (11)	1 (8)	1 (50)

^*^Two patients who underwent CABG received only PLT transfusion for ITP.

PCI, percutaneous coronary intervention; CABG, coronary artery bypass graft; CAD, coronary artery disease; STEMI, ST-elevation myocardial infarction; NSTEMI, non ST-elevation myocardial infarction; UA, unstable angina; PLT, platelet; IVIG, intravenous immunoglobulins; MI, myocardial infarction; ITP, immune thrombocytopenia purpura.

### Characteristics of CAD and ITP cases managed conservatively

Two patients with ITP developed NSTEMI and were managed conservatively without invasive intervention. The decision to use conservative management was attributed to the risk of bleeding in the first case, as the male patient presented with a platelet count of < 10 × 10^9^/L and developed NSTEMI after three doses of IVIG for ITP, while the second case was not revascularized as her NSTEMI was considered to be due to coronary vasospasm, as shown in Table 3, and she did not receive any treatment for ITP. Interestingly, both cases were not prescribed antithrombotic therapy for ACS.

### Outcomes of CAD and ITP cases managed with PCI

Management with PCI was generally safe, as only two out of 18 subjects developed bleeding, which manifested as ecchymosis and diffuse petechiae. As demonstrated in Table 1, one patient died after PCI due to pneumonia. Interestingly, as demonstrated in Table 4, two patients developed STEMI after receiving ITP treatment; one patient developed STEMI within 1 week of IVIG, and the second patient developed STEMI within a few hours of methylprednisolone of 1,000 mg.

### Outcomes of CAD and ITP cases managed with CABG

As demonstrated in Table 2, following CABG, one patient experienced bleeding from the anatomical bed of the internal mammary artery, requiring re-exploration; one patient died within 1 month of surgery due to cardiac tamponade, and one patient developed NSTEMI within 1 day of dexamethasone and rituximab for ITP relapse.

### Outcomes of CAD and ITP cases managed conservatively

The conservative management of ACS did not result in bleeding or mortality events; however, one patient developed NSTEMI after three doses of IVIG for ITP.

## Discussion

The evidence of CAD management in the context of ITP is limited, and such clinical presentation remains very challenging to clinicians as a balance between the risk of bleeding and thrombosis needs to be achieved. Thus, we conducted this systematic review following PRISMA guidelines to summarize the available literature on the management and outcomes of CAD complicated by ITP. Our systematic review included a total of 32 CAD cases that were managed by three different approaches in the setting of ITP: 18 cases had PCI, 12 cases underwent CABG, and the remaining 2 cases were managed conservatively. We have demonstrated that irrespective of intervention used, patients had different CAD presentations, including STEMI, NSTEMI, UA, stable CAD, and a low baseline platelet count, and the majority were treated with either corticosteroids alone, IVIG alone, or a combination of corticosteroids and IVIG. In addition, the antithrombotic therapy regimen was variable, especially among those who underwent PCI. Notably, all CAD treatment modalities resulted in favorable clinical outcomes with respect to bleeding and mortality, and the ITP-induced ACS was relatively very low.

We have demonstrated that, irrespective of CAD presentation, mode of intervention, baseline platelet count, and ITP treatment regimen, patients had favorable clinical outcomes with respect to bleeding and mortality, and the event rate of ACS related to ITP medications was very low.

The management of ACS with thrombocytopenia was addressed in a review by McCarthy et al. in 2017 (34). McCarthy et al. suggested the following strategies to minimize the risk of bleeding among patients with thrombocytopenia and ACS: (1) to proceed with PCI only if platelet count was > 50 x 10^9^/L without active bleeding using radial access instead of femoral access and to use drug-eluting stent (DES) over bare-metal stent (BMS) with limiting DAPT duration to 1 month followed by a single antiplatelet P2Y12 inhibitor, preferably clopidogrel and (2) to hold all anti-platelet agents and avoid PCI in cases where the platelet count was <50 × 10^9^/L or there was concurrent active bleeding. Therefore, the dilemma of ACS management with ITP remains unaddressed and requires further guidance, especially with the presumed thrombotic risks of ITP therapies. The first step in the management of newly diagnosed ITP is to determine whether starting pharmacologic therapy is needed or if close observation of the platelet count is enough. According to the latest guideline for ITP management by the American Society of Hematology (ASH), among those with newly diagnosed ITP and a platelet count of >30 × 10^9^/L with asymptomatic or minor bleeding, observation alone might be used, while for those with a platelet count of <30 × 10^9^/L, pharmacological treatment with corticosteroids is indicated (1). Nevertheless, the challenge with the observation approach among patients with CAD, particularly ACS, is that they require antiplatelet and anticoagulation therapies, which might put them at an increased risk of bleeding if the ITP remains untreated (2, 3).

In our systematic review, we found that patients with symptomatic CAD in the setting of active ITP were treated with different ITP regimens, including corticosteroids alone, IVIG alone, and corticosteroids and IVIG together, in addition to no treatment. According to the latest ASH guidelines for ITP management, the first line therapy for newly diagnosed ITP is a short course of corticosteroids of <6 weeks alone with either prednisone 0.5–2.0 mg/kg/day or dexamethasone 40 mg/day for 4 days as the initial corticosteroid. For those with ITP ≥ 3 months and who are either corticosteroid-dependent or corticosteroid-poor responders, the ASH guidelines suggest using second-line therapy, including TPO-RAs, rituximab, or splenectomy, after appropriate immunizations. Nevertheless, ITP management with ACS remains troublesome, as corticosteroids are associated with bleeding when used with antithrombotic agents and are associated with a possible increased risk of myocardial rupture if used in the setting of ACS (1, 7). Furthermore, second-line therapies are associated with an increased risk of thrombosis (2.5% with thrombopoietin receptor agonists, 2.2% with rituximab, and 2.4% with splenectomy) (1, 8).

Despite the important findings from this systematic review, given the limited literature on co-existing CAD and ITP management, it has a few limitations. First, although we have not restricted our inclusion criteria to any study type, only case reports and case series were identified and included. This highlights the complexity and difficulty of conducting research studies on the vulnerable population of ACS with ITP. Second, we limited our literature search to articles published in English, which might have predisposed the systematic review to language bias and the possibility of missing important findings.

In conclusion, the simultaneous presentation of CAD and ITP presents a significant challenge for clinicians to achieve a sensible balance between the prevention of thrombosis and the bleeding risk. Despite the paucity of evidence of CAD management with ITP, our systematic review of symptomatic CAD in the setting of ITP demonstrated that revascularization with either PCI or CABG with the concurrent use of corticosteroids and IVIG, either alone or in combination, for ITP was feasible with a non-negligible risk of bleeding and mortality. This systematic review may provide reassurance and clinical guidance to cardiologists and hematologists on the feasibility of coronary revascularization in ACS while providing adequate treatment for ITP, and the choice of PCI vs. CABG should be decided depending on the coronary anatomy, severity, and urgency of CAD, baseline platelet count, and patients' concurrent medical conditions and risk factors.

## Data availability statement

The original contributions presented in the study are included in the article/supplementary material, further inquiries can be directed to the corresponding author.

## Author contributions

AR, MY, and DP conceptualized the study. AR and KS did the literature search. AR and AA did the literature screening. KS, MN, AA, and WR extracted the data. AR and KS wrote the first draft of the manuscript. DP, MY, MA-K, MM, MA, and HO edited the manuscript. All authors reviewed the final version of the manuscript. All authors contributed to the article and approved the submitted version.
